# The Rare Sugar Tagatose Differentially Inhibits the Growth of *Phytophthora infestans* and *Phytophthora cinnamomi* by Interfering With Mitochondrial Processes

**DOI:** 10.3389/fmicb.2020.00128

**Published:** 2020-02-06

**Authors:** Abdessalem Chahed, Andrea Nesler, Lorella Navazio, Barbara Baldan, Isabella Busato, Essaid Ait Barka, Ilaria Pertot, Gerardo Puopolo, Michele Perazzolli

**Affiliations:** ^1^Department of Sustainable Agro-Ecosystems and Bioresources, Research and Innovation Centre, Fondazione Edmund Mach, San Michele all’Adige, Italy; ^2^Biological Products for Agriculture (Bi-PA), Londerzeel, Belgium; ^3^Department of Plant Induced Resistance and Bioprotection, University of Reims Champagne-Ardenne, Reims, France; ^4^Department of Biology, University of Padua, Padua, Italy; ^5^Botanical Garden, University of Padua, Padua, Italy; ^6^Center Agriculture Food Environment (C3A), University of Trento, San Michele all’Adige, Italy

**Keywords:** rare sugar, *Phytophthora* spp., biological control, mitochondrial alteration, antioomycete activity, oxidative stress

## Abstract

Rare sugars are monosaccharides with limited availability in nature and their biological functions are largely unknown. Among them, tagatose was developed as a low-calorie sweetener and showed beneficial effects on human health. Tagatose is metabolized by only certain microbial taxa and inhibits the growth of important crop pathogens (e.g., *Phytophthora infestans*), but its mode of action and the microbial responses are unknown. The aim of this study was to understand the tagatose mode of action against *Phytophthora* spp., with the final aim of developing new plant protection products. Tagatose inhibited *P. infestans* growth *in vitro* and caused severe ultrastructural alterations, with the formation of circular and concentric mitochondrial cristae. Decreased ATP content and reduced oxygen consumption rate (OCR) were found in tagatose-incubated *P. infestans* as compared to the control, with the consequent accumulation of reactive oxygen species (ROS) and induction of genes related to apoptosis and oxidative stress response. On the other hand, tagatose did not, or only slightly, affect the growth, cellular ultrastructure and mitochondrial processes in *Phytophthora cinnamomi*, indicating a species-specific response to this rare sugar. The mode of action of tagatose against *P. infestans* was mainly based on the inhibition of mitochondrial processes and this rare sugar seems to be a promising active substance for the further development of eco-friendly fungicides, thanks to its anti-nutritional properties on some phytopathogens and low risk for human health.

## Introduction

Rare sugars are monosaccharides and their derivatives that rarely exist in nature (Granström et al., 2004). The ecological role of rare sugars is not fully understood and their promising biological properties are underestimated, mainly due to their limited availability in terms of quantity in nature (Li et al., 2013). The implementation of industrial enzymatic and microbial processes lowered the cost of rare sugar synthesis (Granström et al., 2004; Izumori, 2006; Oh, 2007) and made scientific studies and technological applications of these carbohydrates more accessible (Oh, 2007; Li et al., 2013). Twenty hexoses (e.g., tagatose, allose, gulose, and sorbose) and nine pentoses (e.g., lyxose, xylulose, and xylitol) have been classified as rare sugars by the international society of rare sugars (Ahmed, 2001; Jayamuthunagai et al., 2017). Among them, tagatose is a ketohexose that was found naturally at low concentration (<3 mg/g) in many foods, such as apples, oranges, and milk (Vastenavond et al., 2011). Tagatose was “generally recognized as safe” by the Food and Drug Administration as it does not have negative impacts on human health (Levin, 2002; Vastenavond et al., 2011). Thanks to its safety for human health, reduced caloric value and physical properties similar to those of sucrose (sweetness, color, and texture), tagatose was approved for use as low-calorie sweetener in several countries, European Union and United States included (Vastenavond et al., 2011).

Tagatose also shows beneficial effects and therapeutic properties on humans and it was proposed for the treatment of “type 2” diabetes, hyperglycemia, anemia, and hemophilia (Levin, 2002). Moreover, tagatose affects the growth of human-associated microorganisms, inhibiting biofilm formation and co-aggregation of the oral bacteria (streptococci and actinomycetes) responsible for dental plaque formation (Levin and Lu, 2007). In particular, prebiotic properties on the human gut microbiome were attributed to tagatose, for example it increases the abundance of beneficial bacteria, such as *Enterococcus* spp. and *Lactobacillus* spp. (Bertelsen et al., 1999; Vastenavond et al., 2011; Hasibul et al., 2018). On the other hand, tagatose inhibits the growth of human pathogenic bacteria, such as *Streptococcus mutants* and *Salmonella enterica* serovar Typhimurium (Lobete et al., 2017; Hasibul et al., 2018). Likewise, tagatose is not catabolized by some human pathogens, such as *Bacillus cereus*, *Escherichia coli*, *Listeria monocytogenes*, *Staphylococcus aureus*, and *Yersinia enterocolitica* (Bautista et al., 2000), indicating its nutritional or anti-nutritional effects on specific microbial taxa. Tagatose can be utilized as a carbohydrate source by only certain microbial taxa, such as *Exiguobacterium* spp., *Lactobacillus* spp., and *Lactococcus* spp. (Raichand et al., 2012; Martinussen et al., 2013; Van Der Heiden et al., 2013; Wu and Shah, 2017). In particular, tagatose can be transported into microbial cells by the phosphotransferase uptake systems and used as an intermediate in the lactose, galactose, and galactitol catabolism by some bacterial species (Van Der Heiden et al., 2013). For example, the *Lactobacillus* spp. and *Lactococcus* spp. metabolism includes the tagatose-6-phosphate pathway (Martinussen et al., 2013; Wu and Shah, 2017) and the incubation of *Lactobacillus rhamnosus* with tagatose triggered a complex transcriptional reprograming of the carbohydrate metabolism with activation of the phosphotransferase system (Koh et al., 2013).

In plants, tagatose inhibits the growth of some phytopathogens and it was patented to control important crop diseases, such as tomato and potato late blight (*P. infestans*), cucumber downy mildew (*Pseudoperonospora cubensis*), grape downy mildew (*Plasmopara viticola*), cucumber powdery mildew (*Sphaerotheca fuliginea*), wheat fusarium blight (*Puccinia recondita*), cabbage downy mildew (*Peronospora parasitica*), rice and cucumber damping-off disease (*Pythium graminicola* and *Pythium aphanidermatum*) (Ohara et al., 2008). Among them, *P. infestans* causes severe economic losses on potato, tomato, and eggplant (Fry et al., 2015) and the *Phytophthora* genus comprises some of the most aggressive and widespread plant pathogens (Kamoun, 2000). For example, *Phytophthora cinnamomi* causes considerable damage to agricultural, horticultural and forest plants, with more than 3000 host species, including avocado, chestnut, and pineapple (Hardham, 2005).

Potato late blight, caused by *P. infestans*, has an estimated cost for growers of about 5 billion dollars per year and requires frequent applications of plant-protection products (Judelson and Blanco, 2005) with a consequent negative impact on human health and the environment (Fantke et al., 2012). Thus, tagatose has been previously suggested as a possible alternative to synthetic chemical fungicides (Ohara et al., 2008), thanks to the absence of deleterious effects on human health (Levin, 2002; Vastenavond et al., 2011). Tagatose showed also possible plant prebiotic effects on the phyllosphere microbiota and modified the balance of potential pathogenic and potential beneficial microorganisms by selective nutritional and anti-nutritional properties for some specific microbial taxa (Perazzolli et al., 2020). However, deeper investigations are required to clarify the growth inhibition properties of tagatose on phytopathogens, because the mechanism of action is still unknown. In addition, tagatose did not inhibit the mycelial growth of *Aspergillus niger*, *Cladosporium cladosporioides*, and *Penicillium chrysogenum* (Izumori et al., 2008) and it promoted the spore germination of *A. niger* (Hayer et al., 2013), indicating the absence of growth inhibition on some plant-associated microorganisms. More specifically, tagatose supported the growth of *Trichoderma harzianum* and *Trichoderma pleuroticola*, but not that of *Trichoderma pleurotum* (Komon-Zelazowska et al., 2007), indicating nutritional or anti-nutritional effects also within species belonging to the same genus. The variability in the response of plant-associated microorganisms to tagatose requires more information on its physiological and molecular effects, to further develop innovative biopesticides based on this active substance. The aim of this study was to clarify the mode of action of tagatose and the cellular responses in two phytopathogenic *Phytophthora* spp. *in vitro*, in order to provide deeper knowledge for the further development of eco-friendly fungicides for sustainable plant protection.

## Materials and Methods

### Biological Material, Growth Conditions and Treatments

*Phytophthora infestans* strain VB3 and *P. cinnamomi* strain CBS 144.22 were stored in glycerol at −80°C in the fungal collection of the Fondazione Edmund Mach, Italy, and they are freely available upon request. *P. infestans* and *P. cinnamomi* were grown in Petri dishes on pea agar medium (PAM, 12.5% frozen peas and 1.2% agar in distilled water) at 18 ± 1 and 25 ± 1°C, respectively (Puopolo et al., 2014).

The *P. infestans* and *P. cinnamomi* mycelial suspension was prepared by collecting small mycelial fragments from 4-days-old colonies. Briefly, Petri dishes of *P. infestans* or *P. cinnamomi* colonies were filled with 2 mL pea broth (PB, 12.5% frozen peas in distilled water), small mycelia fragments were scraped with a sterile spatula and the mycelial suspension was filtered using a sterile Pasteur pipette containing a fine mesh. The liquid culture of *P. infestans* or *P. cinnamomi* was obtained in 10 mL PB inoculated with 100 μL of the mycelial suspension and incubated at 18 ± 1 and 25 ± 1°C under orbital shaking at 80 rpm, respectively.

The stock solution (50 g/L in distilled water) of each rare sugar, such as tagatose (Bi-PA, Londerzeel, Belgium), psicose (Carbosynth, Compton, United Kingdom), and sorbose (Carbosynth), was filter sterilized and added at the appropriate final concentration (5 or 10 g/L) in PAM or PB shortly before *Phytophthora* spp. inoculation. Filter sterilized oligomycin (Sigma-Aldrich, St. Louis, MO, United States) was used as control treatment at the final concentration of 10 μg/mL, since it is known as a growth inhibitor of fungi, such as *Aspergillus* spp., *Candida* spp., and *Penicillium* spp. (Eliskases-Lechner and Prillinger, 1996), through inhibition of ATP synthase activity (Manfredi et al., 2002; Kabala et al., 2014) and mitochondrial respiration (Galloway et al., 2012; Kooragayala et al., 2015).

### Assessment of Rare Sugar Impact on *Phytophthora* spp. Radial Growth

*Phytophthora* spp. plugs (5 mm diameter) were cut from the edge of 14-days-old colonies and a plug was placed at the center of each Petri dish (90 mm diameter) on PAM augmented with the appropriate concentration of the rare sugar. The radial growth of *P. infestans* and *P. cinnamomi* was assessed 4 and 10 days after incubation (DAI) at 18 ± 1 and 25 ± 1°C, respectively, calculated as the average of the two perpendicular diameters of the colony, minus the plug diameter and the result divided by two. Plugs of *P. infestans* and *P. cinnamomi* mycelia developed on PAM in the presence of tagatose were transferred on new PAM dishes and the growth was then monitored as reported above. Ten replicates (dishes) were used for each treatment and the experiment was carried out twice.

### Ultrastructural Analysis by Transmission Electron Microscopy

The *P. infestans* and *P. cinnamomi* liquid cultures were collected at 4 and 10 DAI in PB in the absence (control) and presence of 5 g/L tagatose or 10 μg/mL oligomycin, for transmission electron microscopy (TEM) analysis. Each mycelial sample (0.3 cm^3^) was transferred into a 2 mL tube and incubated with 500 μL of fixing solution (3% glutaraldehyde in 0.1 M cacodylate buffer, pH 7.4) (Zuppini et al., 2010) for 1 h at room temperature under rotary shaking at 15 rpm and then for 15 h at 4°C. Samples were washed three times in 500 μL of cacodylate buffer (0.1 M, pH 7.4), post-fixed for 2 h with 1% (w/v) osmium tetroxide in 0.1 M cacodylate buffer (pH 7.4) and dehydrated in graded ethanol series of 25, 50, 75, and 100% (v/v) with an incubation of 15 min at room temperature for each ethanol concentration (Zuppini et al., 2010). Samples were infiltrated in 1:3 (v/v) araldite resin:propylene oxide (Sigma-Aldrich) by incubating for 1 h at 40°C, followed by 1:1 (v/v) araldite resin:propylene oxide for 1 h at 40°C and 3:1 araldite resin:propylene oxide overnight at 40°C. Samples were subsequently embedded in 100% araldite resin by incubating for 24 h at 40°C and for 72 h at 60°C. Ultra-thin sections (70 nm) were obtained on a Reichert-Jung ultramicrotome (Leica Biosystems, Wetzlar, Germany) and mounted on uncoated copper grids. Sections were then stained with 1% uranyl acetate (in 50% ethanol) for 15 min and 1% lead citrate for 7 min. Observations were carried out with a Tecnai G^2^ transmission electron microscope (Field Electron and Ion Company, Hillsboro, OR, United States) operating at 100 kV and equipped with a Osis Veleta camera (Olympus, Tokyo, Japan). Two replicates (tubes) were analyzed for each treatment and time point and the experiment was carried out twice.

### Assessment of the ATP Content

The *P. infestans* and *P. cinnamomi* mycelial suspension (200 μL) was incubated for 24 h in PB in the absence (control) and presence of 5 g/L tagatose or 10 μg/mL oligomycin in white 96-well microplate with clear flat bottom (Corning, New York, NY, United States) under orbital shaking at 80 rpm at 18 ± 1 and 25 ± 1°C, respectively. Cellular ATP content was quantified using an ATPlite luminescence assay kit (Perkin Elmer, Waltham, MA, United States) according to manufacturer’s instructions (Lis et al., 2016). Briefly, each mycelial suspension was supplemented with 100 μL of lysis buffer under vigorous shaking at 700 rpm for 10 min and 100 μL were then transferred into a 96-well microplate with a transparent flat bottom (Costar, Corning) for the subsequent protein quantification. In each well, 50 μL of substrate solution (luciferin and luciferase) were added after discarding 50 μL of each sample. The luminescence resulting from the reaction of ATP was measured using a Synergy 2 Multi-Mode Microplate Reader (Biotek, Winooski, VT, United States). An ATP standard curve (0.001, 0.01, 0.1, 1, 10, 100, and 1000 nmol/L) was used as reference to calculate the ATP content.

For protein quantification, each sample (100 μL) was mixed with 100 μL Bradford reagent (Pierce Coomassie Plus, Thermo Fisher Scientific), the 96-well microplate was incubated for 10 min at room temperature and the absorbance at 595 nm was measured with a Synergy 2 Multi-Mode Microplate Reader (Biotek). A standard curve of bovine serum albumin (Sigma-Aldrich; 0, 0.01, 0.05, 0.1, and 0.2 mg/mL) was used as reference to determine the protein concentration of each sample and the ATP content was then expressed per unit of total proteins (nmol/mg) (Smith et al., 2016). Three replicates (wells) were assessed for each treatment and the experiment was carried out twice.

### Assessment of the Oxygen Consumption Rate

The *P. infestans* and *P. cinnamomi* mycelial suspension (100 μL) was incubated for 16 h in PB in the absence (control) and presence of 5 g/L tagatose or 10 μg/mL oligomycin in a black 96-well microplate with Corning under orbital shaking at 80 rpm at 18 ± 1 and 25 ± 1°C, respectively. The oxygen consumption rate (OCR) was measured using the MitoXpress Xtra Oxygen Consumption Assay (Luxcel Biosciences, Agilent, Santa Clara, CA, United States) fluorescent probe for the real-time analysis of cellular respiration (Calmes et al., 2015). An aliquot (50 μL) of the liquid media was removed from each well by aspiration with a micropipette and 50 μL of fresh PB containing 100 nM MitoXPress were added and overlaid with 100 μL of mineral oil, to exclude ambient air. Oxygen depletion in the medium was assessed as the increase in the fluorescence lifetime (FLT) of the probe, using a Synergy 2 Multi-Mode Microplate Reader equipped with a time-resolved fluorescence head (Biotek). The relative fluorescence units (RFU) (340 nm excitation, 605–705 nm emission) were recorded twice for 30 μsec, with a delay of 40 μsec (after 30 and 70 μs) at 0, 4, and 8 h of reaction time at 18 ± 1 and 25 ± 1°C for *P. infestans* and *P. cinnamomi* under orbital shaking at 80 rpm, respectively. The FLT of each sample was calculated based on the RFU with 40 μsec delay (Calmes et al., 2015) as follows:

FLT⁢(μ⁢s⁢e⁢c)=(40)/Ln⁢(RFU1/RFU2)

where, 40 μsec is the delay time between the two measurements; RFU1 is the signal measured after 30 μsec and RFU2 is the signal measured after 70 μsec. Three replicates (wells) were assessed for each treatment and the experiment was carried out twice.

### Quantification of Intracellular Reactive Oxygen Species

The *P. infestans* and *P. cinnamomi* mycelial suspension (100 μL) was incubated for 16 h in PB in the absence (control) and presence of 5 g/L tagatose in a black 96-well microplate with Corning under orbital shaking at 80 rpm at 18 ± 1 and 25 ± 1°C, respectively. As control treatment, 2 mM H_2_O_2_ was added to increase reactive oxygen species (ROS) generation, as previously reported for *Aspergillus fumigatus* (Shekhova et al., 2017). Intracellular ROS were quantified with 2′,7′-dichlorodihydrofluorescein diacetate (H_2_DCF-DA; Molecular Probes, Thermo Fisher Scientific) as previously described (Shekhova et al., 2017) with slight modifications. Briefly, 1 μL H_2_DCF-DA (300 μM) was added to each well and the 96-well microplate was incubated for 1 h in the dark under orbital shaking at 80 rpm, at 18 ± 1 and 25 ± 1°C for *P. infestans* and *P. cinnamomi*, respectively. The mycelial suspension was centrifuged at 200 rpm for 2 min, 50 μL of the liquid media were removed by aspiration with a micropipette and replaced with 50 μL of fresh PB to remove the excess of unreacted fluorescent probe. Intracellular ROS were quantified at 0, 1, and 2 h of reaction time by measuring the fluorescence intensity using a Synergy 2 Multi-Mode Microplate Reader (Biotek) with an excitation filter at 485 nm and an emission filter at 530 nm, at 18 ± 1°C for *P. infestans* and at 25 ± 1°C for *P. cinnamomi* (Wong et al., 2018). Three replicates (wells) were assessed for each treatment and the experiment was carried out twice.

### Primer Design for Gene Expression Analysis

The *Phytophthora* spp. gene markers related to sugar metabolism, respiration process, oxidative stress response and apoptosis were selected for quantitative real-time PCR (qPCR) analysis. For each gene, a primer pair compatible for the *P. infestans* and *P. cinnamomi* sequence was designed on conserved coding regions (Supplementary Table S1) and PCR products were sequenced on both strands using an AB3730xl instrument (Applied Biosystems, Thermo Fisher Scientific) at the sequencing platform facility of Fondazione Edmund Mach as validation.

### RNA Extraction and Gene Expression Analysis

The *P. infestans* and *P. cinnamomi* mycelium was collected at 4 and 10 DAI in PAM covered with sterile cellophane layers in the absence (control) and presence of 5 g/L tagatose at 18 ± 1 and 25 ± 1°C, respectively. Samples were immediately frozen in liquid nitrogen, stored at −80°C and crushed using a mixer mill disruptor (MM200, Retsch, Haan, Germany) at 25 Hz for 45 s with sterile steel jars and beads refrigerated in liquid-N_2_. Total RNA was extracted from 100 mg of ground *Phytophthora* spp. mycelium using the Spectrum Plant total RNA kit (Sigma-Aldrich). RNA was quantified by NanoDrop 8000 (Thermo Fisher Scientific, Wilmington, DE, United States), treated with DNase I (Invitrogen, Thermo Fisher Scientific) and the first strand cDNA was synthesized from 1 μg of total RNA using Superscript III (Invitrogen, Thermo Fisher Scientific) and oligo-dT primer. qPCR reactions were carried out with Platinum SYBR Green qPCR SuperMix-UDG (Invitrogen, Thermo Fisher Scientific) and specific primers (Supplementary Table S1) using the Light Cycler 480 (Roche Diagnostics, Mannheim, Germany) as previously described (Perazzolli et al., 2011). Briefly, the PCR conditions were: 50°C for 2 min and 95°C for 2 min as initial steps, followed by 40 cycles at 95°C for 15 s and at 60°C for 1 min. Each sample was examined in three technical replicates and dissociation curves were analyzed to verify the specificity of each amplification reaction. Three housekeeping genes were analyzed, β-tubulin (*tub-b*) (Yan and Liou, 2006), exosome complex exonuclease subunit *Rrp42* and exosome complex exonuclease subunit *Rrp43* (also called exosome ribonuclease) (Judelson et al., 2008), and their stability was validated using the ΔCt method described by Silver et al. (2006). Briefly, a qPCR was carried out for the three housekeeping genes on all samples and *tub-b* was selected as constitutive gene for normalization, because *tub-b* expression was not affected by the treatments (i.e., lowest standard deviation among the housekeeping genes tested). For the gene expression analysis, Light Cycler 480 SV1.5.0 software (Roche) was used to extract Ct values based on the second derivative calculation and the LinReg software version 11.0 was used to calculate reaction efficiencies for each primer pair (Ruijter et al., 2009). The relative expression level (fold change) of each gene was then calculated according to the Pfaffl equation (Pfaffl, 2001) for tagatose-incubated samples as compared to the respective control samples (calibrator) for each time point and *Phytophthora* spp., using *tub-b* as constitutive gene for normalization. Five replicates (dishes with 10 plugs in each dish) were assessed for each treatment and the experiment was carried out twice.

### Statistical Analysis

All experiments were carried out twice and data were analyzed with Statistica 13.1 software (Dell, Round Rock, TX, United States). Normal distribution (Kolmogorov–Smirnov test, *P* > 0.05) and variance homogeneity of the data (Levene’s tests, *P* > 0.05) were checked and parametric tests were used when both assumptions were respected. Each experimental repetition was analyzed singularly and a two-way analysis of variance (ANOVA) was used to demonstrate non-significant differences between the two experiments (*P* > 0.05). Data from the two experimental repetitions were pooled and significant differences were assessed with the Student’s *t*-test (*P* ≤ 0.05) or Tukey’ test (*P* ≤ 0.05) in case of pairwise or multiple comparisons, respectively. Fold change values of gene expression analysis were transformed using the equation *y* = Log_10_ (1 + x) (Casagrande et al., 2011). When parametric assumptions were not respected, the Kruskal–Wallis test was used to demonstrate non-significant differences between the two experimental repetitions (*P* > 0.05), then data from the two experiments were pooled and a Kruskal–Wallis test was used to detect significant differences among treatments (*P* ≤ 0.05).

## Results

### Tagatose Differentially Inhibits *Phytophthora infestans* and *P. cinnamomi* Growth and Causes Ultrastructural Alterations

**FIGURE 1:**
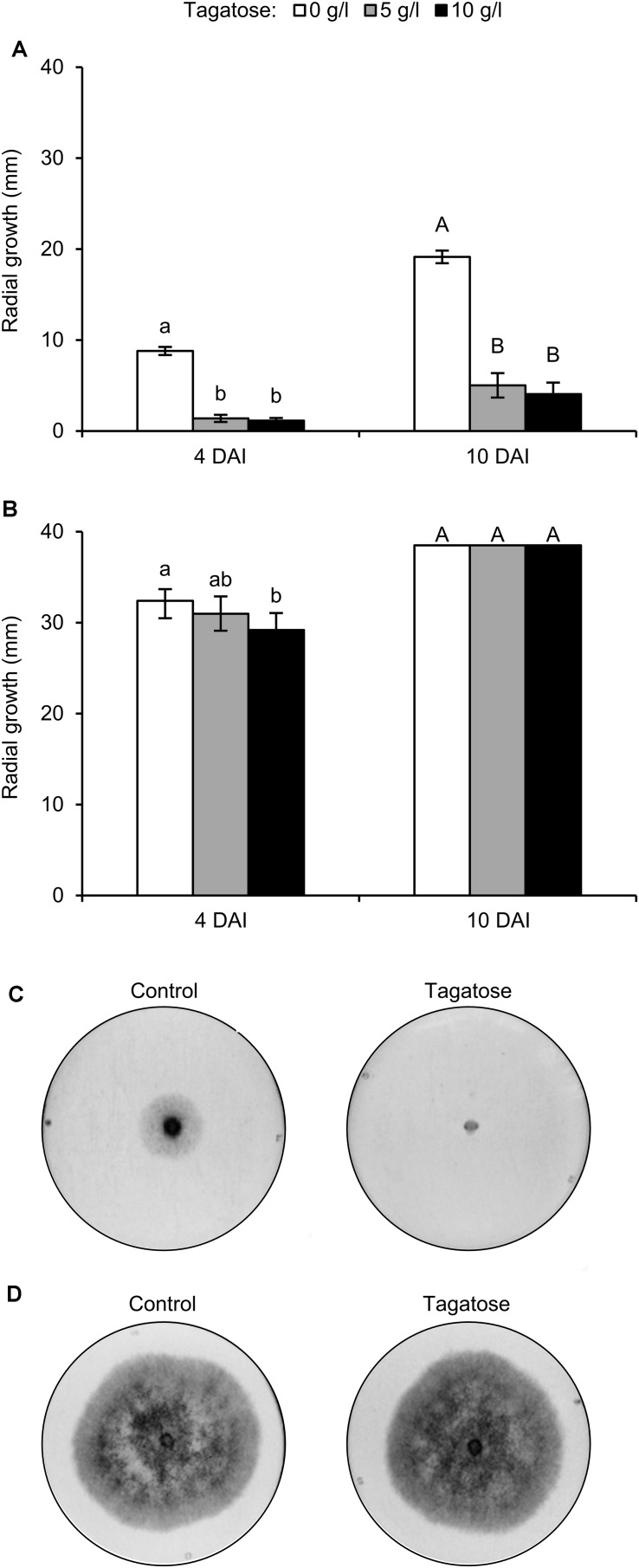
Effect of tagatose on *Phytophthora* spp. growth. *Phytophthora infestans* **(A)** and *P. cinnamomi* **(B)** growth (mm) was assessed 4 and 10 days after incubation (DAI) on pea agar medium in the absence (white, Control) and presence of 5 (gray), or 10 g/L (black) tagatose. The radial growth was calculated as the average of the two perpendicular diameters of the colony, minus the plug diameter and the result divided by two. The Kruskal–Wallis test showed no significant differences between the two experimental repetitions (*P* > 0.05, 10 replicates per experiment) and data from the two experiments were pooled. Mean and standard error values of 20 replicates (dishes) from the two experiments are presented for each treatment. Different lowercase and uppercase letters indicate significant differences among treatments at 4 and 10 days after incubation (DAI) according to the Kruskal–Wallis test (*P* ≤ 0.05), respectively. Representative pictures of *P. infestans* **(C)** and *P. cinnamomi* **(D)** growth 4 DAI in the absence (Control) and presence of 5 g/L tagatose are shown.)

*Phytophthora infestans* growth was inhibited by tagatose at 4 and 10 DAI and the level of inhibition using 5 and 10 g/L tagatose was comparable at each time point (Figure 1A). Conversely, *P. cinnamomi* growth was not affected by 5 and 10 g/L tagatose at 10 DAI, and only a slight inhibition was observed with 10 g/L tagatose at 4 DAI (Figure 1B). When transferred to new PAM dishes, the growth of *P. infestans* plugs collected at 10 DAI with 5 and 10 g/L tagatose was comparable to the growth of *P. infestans* plugs collected at 10 DAI from control dishes (data not shown), indicating that the effect of tagatose was reversible.

In order to verify the specificity of tagatose, isomers were tested, such as fructose, psicose, and sorbose. The incubation with 5 g/L fructose, 5 g/L psicose, or 5 g/L sorbose did not inhibit *P. infestans* and *P. cinnamomi* growth at 4 DAI (Supplementary Figure S1) and 10 DAI (data not shown). Because the minimum dosage showing a differential effect on *Phytophthora* spp. growth was 5 g/L tagatose (Figures 1C,D), this quantity was selected for the following experiments.

**FIGURE 2:**
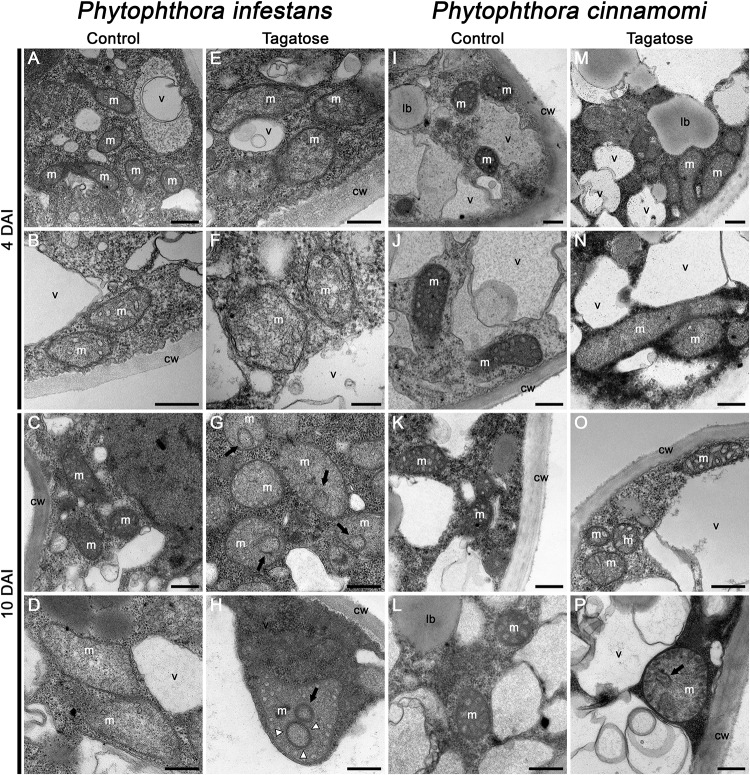
Effect of tagatose on *Phytophthora* spp. ultrastructure. Transmission electron microscopy observations of *P. infestans* **(A–H)** and *P. cinnamomi* **(I–P)** liquid culture were carried out 4 and 10 days after incubation (DAI) in pea broth either in the absence (Control) or the presence of 5 g/L tagatose. Two replicates (tubes) were analyzed for each treatment and the experiment was carried out twice with similar results. Circular (black arrows) and concentric (white arrowheads) mitochondrial cristae are indicated. cw, cell wall; lb, lipid bodies; m, mitochondria; v, vacuoles. Bars correspond to 500 nm **(A–E, G–P)** or 200 nm **(F)**. Two representative pictures are reported for each treatment and time point as follow: **A,B**: *P. infestans* control 4 DAI; **C,D**: *P. infestans* control 10 DAI; **E,F**: *P. infestans* tagatose 4 DAI; **G,H**: *P. infestans* tagatose 10 DAI; **I,J**: *P. cinnamomi* control 4 DAI; **K,L**: *P. cinnamomi* control 10 DAI; **M,N**: *P. cinnamomi* tagatose 4 DAI; **O,P**: *P. cinnamomi* tagatose 10 DAI.

In order to investigate morphological impacts of tagatose incubation on cellular structures, TEM analyses were carried out. The typical ultrastructure of *Phytophthora* spp. (Xu et al., 2007) was observed by TEM analysis of *P. infestans* collected at 4 DAI (Figures 2A,B) and 10 DAI in PB (Figures 2C,D). Conversely, the structure of mitochondria was severely altered in tagatose-incubated *P. infestans* samples, displaying the reorganization of mitochondrial cristae at 4 DAI (Figures 2E,F) that leads to circular and concentric cristae at 10 DAI (Figures 2G,H). In the case of *P. cinnamomi*, tagatose did not affect cellular structures at 4 DAI (Figures 2M,N) as compared to the control (Figures 2I–L). Slight mitochondrial alterations, consisting of a less dense mitochondrial matrix with a sporadic occurrence of circular cristae, were found in *P. cinnamomi* only at 10 DAI (Figures 2O,P). The ATP synthase inhibitor oligomycin (Manfredi et al., 2002; Kabala et al., 2014), known to impair fungal growth (Eliskases-Lechner and Prillinger, 1996) and mitochondrial respiration (Galloway et al., 2012; Kooragayala et al., 2015), caused severe mitochondrial alterations in both *P. infestans* and *P. cinnamomi* already at 4 DAI and showed either the disappearance or profound rearrangement of the mitochondrial cristae (Supplementary Figure S2). Taken together, these observations indicated that tagatose altered the mitochondrial structure of *P. infestans* with consequent inhibition of radial growth.

### Tagatose Negatively Affects Mitochondrial Activities in *Phytophthora infestans* and Not *P. cinnamomi*

**FIGURE 3:**
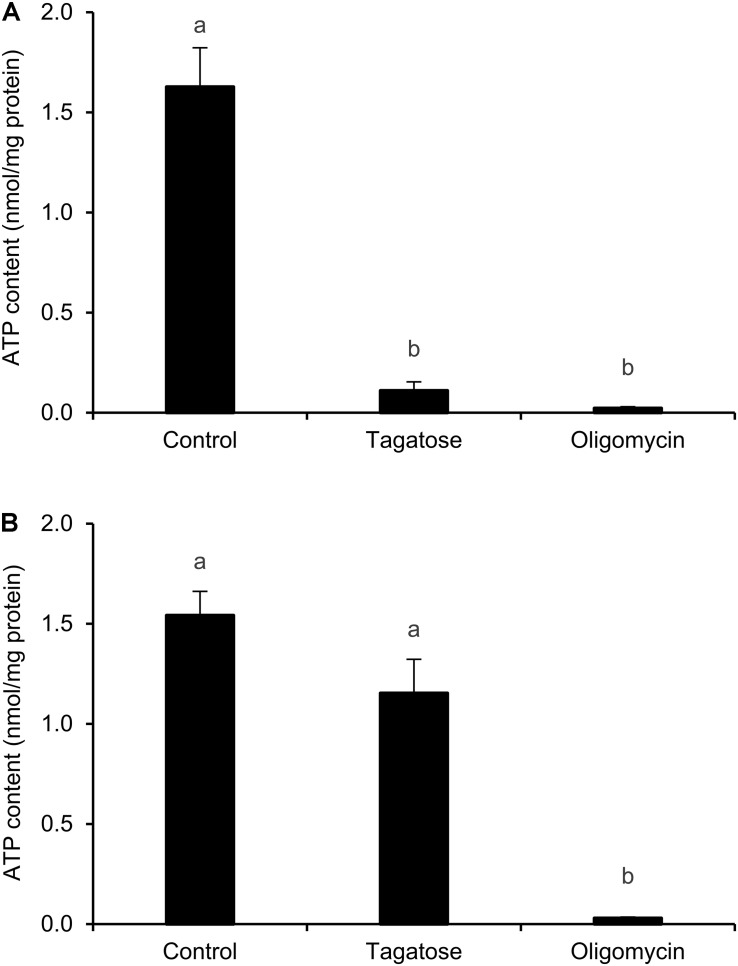
Effect of tagatose on ATP content of *Phytophthora* spp. The ATP content of *P. infestans* **(A)** and *P. cinnamomi* **(B)** mycelial suspension was assessed 24 h after incubation in pea broth in the absence (Control) and presence of 5 g/L tagatose or 10 μg/mL oligomycin. The ATP content was quantified using the luminescence assay and expressed per unit of total proteins (nmol/mg). The Kruskal–Wallis test showed no significant differences between the two experimental repetitions (*P* > 0.05, three replicates per experiment) and data from the two experiments were pooled. Mean and standard error values of six replicates (wells) from the two experiments are presented for each treatment. Different letters indicate significant differences among treatments according to the Kruskal–Wallis test (*P* ≤ 0.05).

Since mitochondrial cristae alterations have been associated with the dysfunction of ATP synthase activity in yeast (Paumard et al., 2002; Gavin et al., 2004; Weimann et al., 2008), the ATP content of *Phytophthora* spp. was assessed. Tagatose decreased the ATP content of *P. infestans* as compared to the control and the effect was comparable to that obtained with the ATP synthase inhibitor oligomycin (Figure 3A). The ATP content of *P. cinnamomi* was not affected by tagatose and it was decreased by only the ATP synthase inhibitor oligomycin (Figure 3B).

**FIGURE 4:**
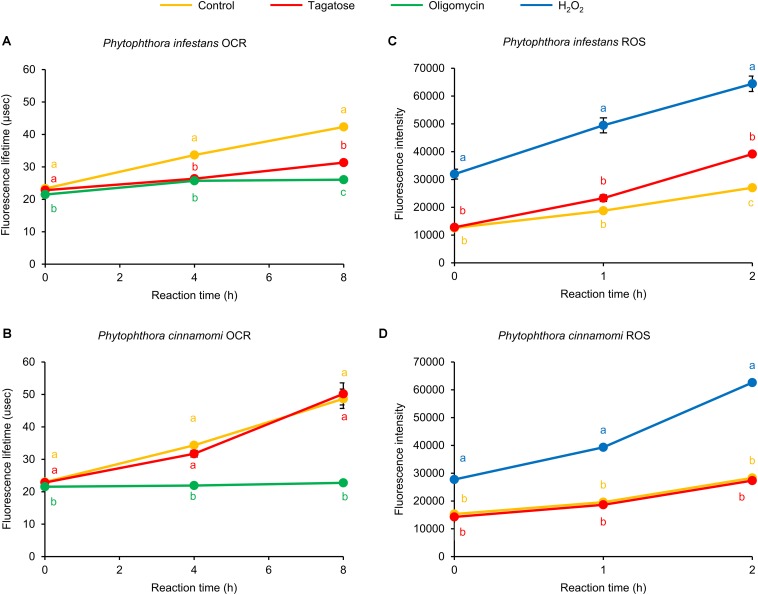
Effect of tagatose on *Phytophthora* spp. respiration processes and accumulation of reactive oxygen species (ROS). The oxygen consumption rate (OCR) and the generation of ROS of the *P. infestans* **(A,C)** and *P. cinnamomi* **(B,D)** mycelial suspension were assessed 16 h after incubation in pea broth in the absence (Control, orange) and presence of 5 g/L of tagatose (red). Oligomycin (10 μg/mL, green) and hydrogen peroxide (2 mM H_2_O_2_, blue) were used as control treatments of the OCR and ROS assay, respectively. The OCR was assessed as increase of fluorescence lifetime (μsec) at 0, 4, and 8 h of reaction time and ROS generation was quantified as fluorescence intensity at 0, 1, and 2 h of reaction time using fluorescent probes. For each assay, the factorial analysis of variance showed no significant differences between the two experimental repetitions (*P* > 0.05, three replicates per experiment) and data from the two experiments were pooled. Mean and standard error values of six replicates (wells) from the two experiments are presented for each treatment. Different letters indicate significant differences among treatments, according to the Tukey test (*P* ≤ 0.05).

The ATP synthase inhibition has been commonly linked to dysfunctions of the OCR (Galloway et al., 2012; Kooragayala et al., 2015) and ROS homeostasis (Martinez-Reyes and Cuezva, 2014) of eukaryotic cells. In *P. infestans*, we found that the OCR was inhibited by tagatose at 4 and 8 h reaction time and the FLT of tagatose-incubated samples was intermediate between that of control samples and samples incubated with the ATP synthase inhibitor oligomycin (Figure 4A). Conversely, *P. cinnamomi* OCR was not affected by tagatose and it was impaired by only the ATP synthase inhibitor oligomycin (Figure 4B). As possible consequence of ATP synthase and OCR inhibition, the ROS generation was increased by tagatose in *P. infestans* (Figure 4C), but not in *P. cinnamomi* (Figure 4D). As for other systems (Shekhova et al., 2017), H_2_O_2_ incubation increased the ROS level in both *Phytophthora* spp. In particular, the ROS fluorescence intensity of tagatose-incubated *P. infestans* was intermediate between that of control and H_2_O_2_-incubated samples. Taken together, these results showed that tagatose decreased the ATP content and OCR with a consequent increase of ROS accumulation in *P. infestans*, but not in *P. cinnamomi*.

### Tagatose Modulates the Expression of *Phytophthora* spp. Genes

*Phytophthora* spp. genes encoding key enzymes of glycolysis were analyzed by qPCR (Supplementary Table S1), such as glucose-6-phosphate dehydrogenase (*g6pd*) and phosphofructokinase (*pfk*). Moreover, genes encoding ATP synthase subunits, responsible for ATP production (Yoshida et al., 2001) were selected, since their activity was previously linked to mitochondrial structure biogenesis in *Saccharomyces cerevisiae* (Paumard et al., 2002; Lefebvre-Legendre et al., 2005), such as the ATP synthase subunit 4 (*atp4*) and ATP synthase subunit beta (*atpB*). Maleylacetoacetate isomerase (*maai*) was analyzed and it shares sequence homology and key domains with glutathione S-transferase genes (*gst*) upregulated by oxidative stresses (Montibus et al., 2015). The apoptosis-inducing factor (*aif*) was analyzed as marker of ROS scavenging (Klein et al., 2002) and mitochondrial cristae regulation (Cheung et al., 2006), since it was upregulated in response to farnesol in *Aspergillus nidulans* (Savoldi et al., 2008). The pro-apoptotic serine protease (*nma111*) and cytochrome c (*cytc*) were selected as upregulated genes in response to graphene oxide (Zhu et al., 2017) and acetic acid in *S. cerevisiae* (Ludovico et al., 2002), respectively. Possible markers of oxidative phosphorylation were selected, such as genes encoding NADH dehydrogenase ubiquinone flavoprotein (*ndufv*) (Kuhn et al., 2015) and cytochrome c oxidase (*cox*) (Dufour et al., 2000), as well as the cellulose synthase (*ces*) responsible for cell wall biosynthesis (Blum et al., 2010).

Expression levels of genes related to apoptosis (*aif* and *nma111*) and oxidative stress response (*maai*) were upregulated by tagatose in *P. infestans* at 4 and 10 DAI (Table 1). The *atp4* expression was upregulated by tagatose at 4 DAI, suggesting an attempted *P. infestans* response to the ATP decrease and ROS increase caused by tagatose. On the other hand, the expression of *atpB*, *ces*, *cytc*, *cox*, *g6pd*, *ndufv*, and *pfk* was not affected by tagatose in *P. infestans*. In *P. cinnamomi*, no genes of glycolysis (*g6pd* and *pfk*), ATP synthesis (*atp4* and *atpB*), apoptosis (*aif*), oxidative phosphorylation (*ndufv* and *cox*), cellulose biosynthesis (*ces*), and oxidative stress response (*maai* and *cytc*) were modulated by tagatose at 4 DAI and 10 DAI, except for the *nma111* downregulation at 10 DAI, as corroboration of slight tagatose effects on *P. cinnamomi*.

**TABLE 1:** Effect of tagatose on *Phytophthora* spp. gene expression.

**Gene description**	**Abbreviation**	***Phytophthora infestans***		***Phytophthora cinnamomi***	
			
		**4 DAI**	**10 DAI**	**4 DAI**	**10 DAI**
Apoptosis inducing factor mitochondria associated	*aif*	1.8 ± 0.1*	1.5 ± 0.1*	1.1 ± 0.1	−1.3 ± 0.3
ATP synthase subunit 4	*atp4*	1.5 ± 0.1*	1.4 ± 0.1	1.1 ± 0.1	1.1 ± 0.0
ATP synthase subunit beta	*atpB*	1.2 ± 0.2	−0.9 ± 0.0	1.0 ± 0.1	1.1 ± 0.1
Cellulose synthase	*ces*	1.3 ± 0.2	1.1 ± 0.0	−1.3 ± 0.1	−1.8 ± 0.1
Cytochrome c	*cytc*	1.0 ± 0.1	−1.2 ± 0.1	1.0 ± 0.2	−1.4 ± 0.3
Cytochrome c oxidase	*cox*	1.4 ± 0.6	1.6 ± 0.6	−1.3 ± 0.1	1.2 ± 0.0
Glucose-6-phosphate dehydrogenase	*g6pd*	1.1 ± 0.1	1.2 ± 0.2	1.0 ± 0.1	1.0 ± 0.4
Maleylacetoacetate isomerase	*maai*	5.8 ± 0.4*	8.3 ± 1.1*	1.1 ± 0.2	−1.2 ± 0.6
NADH dehydrogenase ubiquinone flavoprotein	*ndufv*	−1.1 ± 0.1	1.3 ± 0.1	1.0 ± 0.3	−1.1 ± 0.2
Phosphofructokinase	*pfk*	1.2 ± 0.1	1.3 ± 0.1	1.0 ± 0.2	−1.3 ± 0.2
Pro-apoptotic serine protease nma111-like protein	*nma111*	2.0 ± 0.3*	2.0 ± 0.2*	−1.3 ± 0.2	−3.2 ± 0.1*

*Relative expression levels (fold changes) of *P. infestans* and *P. cinnamomi* target genes (Supplementary Table S1) were assessed by quantitative real-time PCR 4 and 10 days after incubation (DAI) on PAM in the absence (Control) and presence of 5 g/L tagatose. The two-way analysis of variance (ANOVA) revealed no significant differences between the two experimental repetitions (*P* > 0.05, five replicates per experiment) and data from the two experiments were pooled. Mean fold change and standard error values of 10 replicates (dishes) from the two experiments are presented for tagatose-incubated samples as compared to the respective control samples, using β-tubulin as a constitutive gene for normalization. Asterisks indicate significant gene upregulation and downregulation in tagatose-incubated samples as compared to the respective control samples with a fold change greater than 1.5 according to the Student’s *t*-test (*P* ≤ 0.05).*

## Discussion

Tagatose is a rare sugar that can be metabolized by only certain microbial taxa (Raichand et al., 2012; Martinussen et al., 2013; Van Der Heiden et al., 2013; Wu and Shah, 2017) and inhibits some important crop pathogens, *P. infestans* included (Ohara et al., 2008). Nutritional and anti-nutritional effects of tagatose have been shown on human-associated microorganisms (Van Der Heiden et al., 2013; Lobete et al., 2017; Wu and Shah, 2017; Hasibul et al., 2018) and plant-associated microorganisms (Komon-Zelazowska et al., 2007; Izumori et al., 2008; Ohara et al., 2008; Hayer et al., 2013; Perazzolli et al., 2020). We showed that tagatose inhibited the growth of *P. infestans*, but it had only slight effects on *P. cinnamomi*, with a species-specific impact on the mitochondrial processes. *P. infestans* and *P. cinnamomi* were grown at the respective optimum temperature commonly used for fungicide assays *in vitro* (Coffey and Joseph, 1985; Groves and Ristaino, 2000; Yuan et al., 2006; Hu et al., 2010) and the contribution of temperature to the differential effect of tagatose cannot be totally excluded. The selectivity of action was previously observed on *Trichoderma* spp., where tagatose supported the growth of *T. harzianum* and *T. pleuroticola*, but not that of *T. pleurotum* (Komon-Zelazowska et al., 2007). Similar selectivity was reported in some bacterial genera, for example tagatose can be assimilated by *Bacillus licheniformis* (Van Der Heiden et al., 2013), *Lactobacillus plantarum*, *Lactobacillus acidophilus*, *Lactobacillus brevis* (Bautista et al., 2000), *Lactobacillus casei*, and *L. rhamnosus* (Koh et al., 2013), but not by *B. cereus*, *Bacillus subtilis*, and *Lactobacillus buchneri* (previously called *Lactobacillus frigidus*) (Bautista et al., 2000). Although all the rare sugars tested in this work were epimers (psicose, sorbose, and tagatose), only tagatose inhibited *P. infestans* growth, suggesting that structural differences among epimers may affect the inhibitory activities of rare sugars. The inhibition of *P. infestans* growth was reversible and tagatose-incubated plugs can normally grow when transferred on a new growth medium free of tagatose. This reversible effect raises the question of how to keep the persistence of tagatose on treated crops at constant and sufficient levels, when looking at a possible tagatose application as a plant protection product, and suggest that appropriated formulations should be probably developed for its application under field conditions.

Severe mitochondrial alterations with concentric cristae were found in tagatose-incubated *P. infestans*. Similar alterations have been previously observed in mammalian (Florea and Craciun, 2011) and yeast (Arselin et al., 2004) cells treated with inhibitors of mitochondrial activities, such as *Apis mellifera* venom and doxycycline, respectively. Likewise, some toxic compounds are known to form mitochondrial concentric cristae in target organisms, such as a phosphocholine derivative in *Leishmania amazonensis* (Godinho et al., 2013), benzimidazole anthelmintic in *Haemonchus contortus* (Cristina et al., 2015), and ethidium bromide in green *Euglena* spp. cells (Nass and Ben-Shaul, 1973). Moreover, xylitol and sorbose caused structural alterations of *Coprinus lagopus* cell wall (Moore and Stewart, 1972), *Streptococcus mutans* cell membrane (Nayak et al., 2014), and *Neurospora crassa* vesicle number and size (Trinci and Collinge, 1973), respectively. Mitochondrial cristae alterations were previously associated with the dysfunction of ATP synthase activity (Paumard et al., 2002; Gavin et al., 2004; Weimann et al., 2008) and with the reduction of mitochondrial bioenergetic status (Zick et al., 2009) in yeast cells. In particular, the formation of concentric cristae was associated with dimerization and oligomerization disorders of the ATP synthase in *S. cerevisiae* (Zick et al., 2009) with the consequent uncontrolled biogenesis of the inner mitochondrial membrane (Velours et al., 2009). The ATP synthase dimerization is associated to disulfide bonds between subunits codified by *atp4* in *S. cerevisiae* (Paumard et al., 2002) and the upregulation of *atp4* in *P. infestans* suggested an attempted cellular response against tagatose, in order to mitigate ATP4 dimerization effects. As consequence of mitochondrial alterations, the ATP content was decreased by tagatose in *P. infestans*, as previously reported in human subjects exposed to tagatose (Buemann et al., 2000) and isolated perfused liver treated with xylitol (Woods and Krebs, 1973). The ATP synthase inhibition has been commonly linked to the OCR reduction (Galloway et al., 2012; Kooragayala et al., 2015) and cellular redox state alteration (Martinez-Reyes and Cuezva, 2014), indicating that OCR inhibition and ROS increase in tagatose-incubated *P. infestans* can be ascribed to severe inhibition of mitochondrial processes.

The attempted cellular responses of *P. infestans* against tagatose included the upregulation of *maai*, which is homologous to *gst* genes commonly upregulated by oxidative stresses (Montibus et al., 2015). The *maai* gene is a marker of stress responses and its expression was also upregulated by the biocontrol agent *Lysobacter capsici* AZ78 in *P. infestans* (Tomada et al., 2017) and by copper sulfate in *Saprolegnia parasitica* (Hu et al., 2016). In addition, tagatose upregulated the expression of *aif* in *P. infestans* and it encoded a protein involved in ROS scavenging (Klein et al., 2002) and mitochondrial cristae regulation (Cheung et al., 2006) in mammalian cells. The expression of *nma111* was upregulated and downregulated by tagatose in *P. infestans* and *P. cinnamomi*, respectively, and its expression was previously linked to ROS accumulation in *Saccharomyces* spp. (Wang et al., 2014), demonstrating a strong connection of physiological effects and transcriptional changes observed in tagatose-incubated *P. infestans* and *P. cinnamomi.* Therefore, further transcriptomic and metabolomic studies are required, in order to better understand the species-specific response of *Phytophthora* spp. to tagatose incubation and to estimate the risk of evolution of *P. cinnamomi*-like tolerance to tagatose in *P. infestans*.

## Conclusion

Tagatose inhibited *P. infestans* growth *in vitro* and caused severe ultrastructural alterations, with the formation of circular and concentric mitochondrial cristae. In addition, the ATP content and OCR were decreased, while the ROS accumulation and expression of apoptosis- and oxidative stress-related genes were increased, suggesting the induction of severe deficiencies in the mitochondrial processes of tagatose-incubated *P. infestans*. On the other hand, *P. cinnamomi* growth and mitochondrial ultrastructure were only slightly affected by tagatose with no significant impacts on respiration processes and ROS accumulation, suggesting species-specific responses to this rare sugar. A partial or total selectivity of a fungicide is commonly regarded as a positive trait, because of the reduction of possible side effects on other microorganisms. For this reason, besides the good toxicological profile, tagatose seems to be a promising active substance for the further development of plant protection products to control *P. infestans*. The mode of action of tagatose against *P. infestans* is mainly based on the inhibition of mitochondrial processes, but further transcriptomic and metabolomic analyses are required to fully clarify the molecular determinants and pathways affected by this rare sugar in *Phytophthora* spp. In addition, its reversible effects suggest that efficacy trials of formulated products under field conditions are required, in order to better verify the stability and persistence of tagatose against target phytopathogens.

## Data Availability Statement

All datasets generated for this study are included in the article/Supplementary Material.

## Author Contributions

AC carried out the functional experiments and wrote the manuscript. AN carried out the functional experiments. LN and BB carried out ultrastructural analysis and revised the manuscript. IB carried out growth experiments and an ultrastructural analysis. EA revised the manuscript. IP revised the manuscript and analyzed the data. GP conceived the study, analyzed the data and revised the manuscript. MP conceived the study, supervised the experiments, analyzed the data and wrote the manuscript.

## Conflict of Interest

AC and AN were employed by Biological Products for Agriculture (Bi-PA). The remaining authors declare that the research was conducted in the absence of any commercial or financial relationships that could be construed as a potential conflict of interest.
