# Supplementary material for: The Rare Sugar Tagatose Differentially Inhibits the Growth of Phytophthora infestans and Phytophthora cinnamomi by Interfering With Mitochondrial Processes
Source: Front Microbiol. 2020 Feb 6;11:128. doi: 10.3389/fmicb.2020.00128 (PMC7015900; doi:10.3389/fmicb.2020.00128)
Supplement: Supplementary file 1 [file Data_Sheet_1.PDF]

Supplementary materials

The rare sugar tagatose differentially inhibits the growth of *Phytophthora infestans* and *Phytophthora cinnamomi* by interfering with mitochondrial processes

Abdessalem Chahed[1,2,3], Andrea Nesler[1,2], Lorella Navazio[4,5], Barbara Baldan[4,5], Isabella Busato[1,4], Essaid Ait Barka[3], Ilaria Pertot[1,6], Gerardo Puopolo[1,6] and Michele Perazzolli[1,6,*]

[1] Department of Sustainable Agro-ecosystems and Bioresources, Research and Innovation Centre, Fondazione Edmund Mach, Via E. Mach 1, 38010 San Michele all'Adige, Italy,[2] Biological Products for Agriculture, Bi-PA, Technologielaan 7, 1840 Londerzeel, Belgium, [3] Department of Plant Induced Resistance and Bioprotection, University of Reims, Moulin de la Housse, 51687 Reims, France, [4] Department of Biology, University of Padova, Via U. Bassi 58/B, 35131 Padova, Italy, [5] Botanical Garden, University of Padova, Via Orto Botanico 15,35123 Padova, Italy, [6]Center Agriculture Food Environment (C3A), University of Trento, via E. Mach 1, 38010 San Michele all'Adige, Italy

[*] Correspondence:

Michele Perazzolli, michele.perazzolli@unitn.it

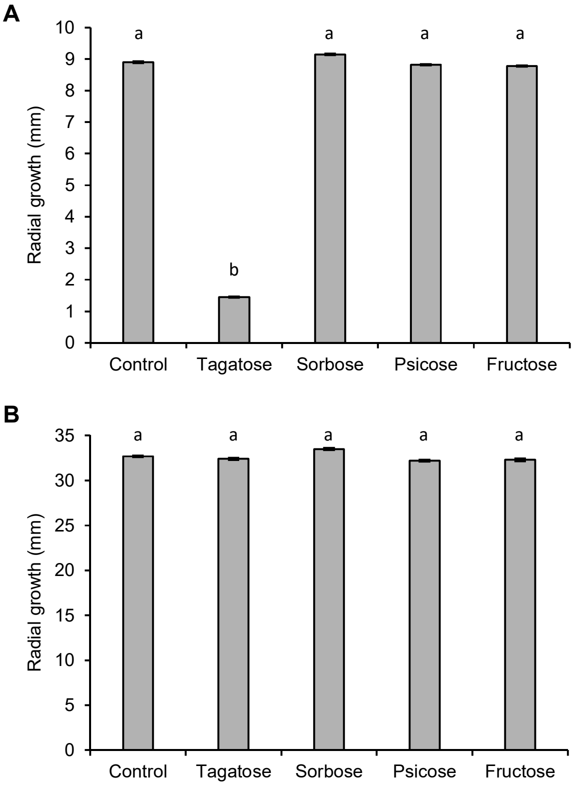

FIGURE S1 | Effect of tagatose isomers on *Phytophthora* spp. growth. *Phytophthora infestans* (**A**) and *P. cinnamomi* (**B**) growth (mm) was assessed four days after incubation on pea agar medium in the absence (control) and presence of 5 g/L tagatose, 5 g/L sorbose, 5 g/L psicose or 5 g/L fructose. The radial growth was calculated as the average of the two perpendicular diameters of the colony subtracted by the plug diameter and divided by two. The Kruskal-Wallis test showed no significant differences between the two experimental repetitions ($P > 0.05$, ten replicates per experiment) and data from the two experiments were pooled. Mean and standard error values of twenty replicates (dishes) from the two experiments are presented for each treatment. Different letters indicate significant differences among treatments according to the Kruskal- Wallis test ($P \leq 0.05$).

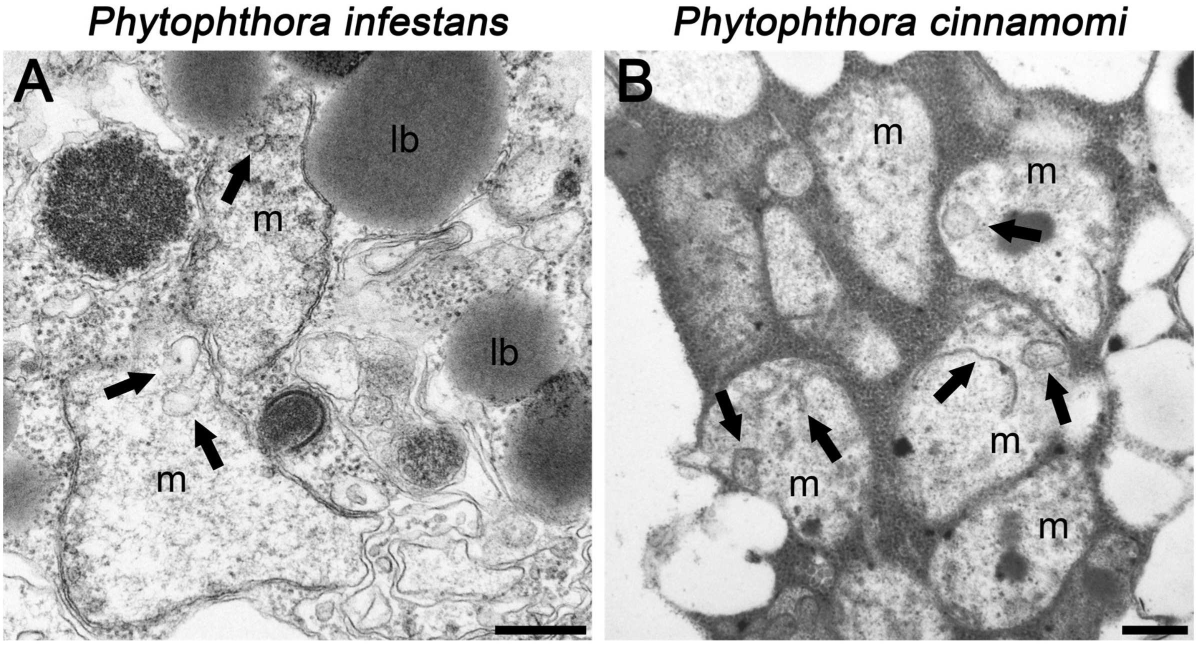

FIGURE S2 | Effect of oligomycin on *Phytophthora* spp. ultrastructure. Transmission electron microscopy (TEM) observations of *P. infestans* (**A**) and *P. cinnamomi* (**B**) liquid cultures were carried out four days after incubation (DAI) in pea broth in the presence of 10 µg/mL oligomycin. Profound rearrangement of mitochondrial cristae (arrows) is indicated. At 10 DAI the ultrastructural organization of the fungal mycelia was deeply compromised and did not allow adequate TEM observations. Two replicates (tubes) were analysed for each treatment and the experiment was carried out twice with similar results. A representative picture is reported for each treatment. Abbreviations: lb, lipid bodies; m, mitochondria. Bars correspond to 500 nm.

Table S1 | Primer sequences of *Phytophthora* spp. genes analysed by quantitative real-time PCR.

Gene Name[1]	Gene abbreviation[2]	*P. infestans* accession number[3]	*P. cinnamomi* accession number[4]	Primer forward (5'-3')[5]	Primer reverse (5'-3')6
Apoptosis inducing factor	*aif*	PITG_21182	PHYCI_216731	GAGCGTGGTGTGGTGTACTA	GTGTGCTTGAGTTCCTCCA
ATP synthase subunit 4	*atp4*	PITG_00074	PHYCI_93991	AGAAGGGCGACAAGAAGC	TGAAGAGAGCAGCCACGT
ATP synthase subunit beta	*atpB*	PITG_06595	PHYCI_116704	AGGTGTTCACGGGCAAG	AACCATGTAGAAGGCAGCC
Cellulose synthase	*ces*	PITG_16984	PHYCI_23931	TACTTCGCCTCGCTGTTC	CTGGTCCGTCAAACTCTTGT
Cytochrome c	*cytc*	PITG_12682	PHYCI_98125	ACCCTGTTCGAGTACCTCCT	TCCATCAGGTAAGCGATCAG
Cytochrome c oxidase	*cox*	808896	KC855438.1	AGCAACTTTATGGGGAGGTT	AGCTACACCAGTTACTCCGC
Glucose-6-phosphate dehydrogenase	*g6pd*	PITG_00146	PHYCI_79086	TGATCCTGGACGTGCTG	AGCGGCTTCACCTTCTG
Maleylacetoacetate isomerase	*maai*	PITG_05380	PHYCI_9126	GCTCGTAGTGGCTTGGAAC	CAGCAGATACACGTCCGC
NADH dehydrogenase ubiquinone flavoprotein	*ndufv*	PITG_06815	PHYCI_323789	ATCCGCAAGTTCAAGCAC	ATTTGTAAGCCGAGCCGT
Phosphofructokinase	*pfk*	PITG_11459	PHYCI_15899	CCAGGATCTCAAGGTGGA	GTCGGGTTTCTTGCAGG
Pro-apoptotic serine protease nma111	*nma111*	PITG_21602	PHYCI_209286	CCAAAATCTACGACCTGGG	ACACTCAAGATAGTTCGGCG
β-tubulin	*tub-b*	PITG_00156	PHYCI_418545	GCTGAGTCCAACATGAACG	CTCATCCATCTCCTCGTCC
Exosome complex exonuclease (subunit Rrp42)	*exos42*	PITG_16138	PHYCI_94282	CAGCAAGATCGGTGACTACTT	GCATCTCCTCCATCTCGA
Exosome complex exonuclease (subunit Rrp43)	*exos43*	PITG_07829	PHYCI_99129	CTCTCGTACAGTACCGACGG	GTTGAAGCGTCCACAAGC

[1] Description of *Phytophthora infestans* and *P. cinnamomi* genes selected for quantitative real-time PCR (Gene Name).
[2] Gene abbreviations were based on annotation of the homologous protein by blastp search against the SwissProt database.
[3] Accession number of *P. infestans* from ENSEMBL (https://protists.ensembl.org/Phytophthora_infestans/Info/Index) and NCBI (https://www.ncbi.nlm.nih.gov/search/) database.
[4] Accession number of *P. cinnamomi* from FungiDB (https://fungidb.org/fungidb/) and NCBI (https://www.ncbi.nlm.nih.gov/search/) database.
[5] Forward and reverse primer sequences used for quantitative real-time PCR.
[6] Genes encoding β-tubulin and exosome complex exonuclease (subunit Rrp42 and subunit Rrp43) were used as constitutive genes for quantitative real-time PCR data normalization.
